# Comparative Transcriptome Analysis of Waterlogging-Sensitive and Waterlogging-Tolerant *Chrysanthemum morifolium* Cultivars under Waterlogging Stress and Reoxygenation Conditions

**DOI:** 10.3390/ijms19051455

**Published:** 2018-05-14

**Authors:** Nan Zhao, Chuanwei Li, Yajun Yan, Wen Cao, Aiping Song, Haibin Wang, Sumei Chen, Jiafu Jiang, Fadi Chen

**Affiliations:** Key Laboratory of Landscape Agriculture, Ministry of Agriculture, College of Horticulture, Nanjing Agricultural University, Nanjing 210095, China; 2015204028@njau.edu.cn (N.Z.); 2017104092@njau.edu.cn (C.L.); 2016104096@njau.edu.cn (Y.Y.); 14114226@njau.edu.cn (W.C.); aiping_song@njau.edu.cn (A.S.); hb@njau.edu.cn (H.W.); chensm@njau.edu.cn (S.C.); jiangjiafu@njau.edu.cn (J.J.)

**Keywords:** waterlogging, reoxygenation, *Chrysanthemum morifolium*, transcriptome, ethylene, transcription factors

## Abstract

Waterlogging stress is among the most severe abiotic stressors in the breeding and the production of *Chrysanthemum morifolium.* However, the mechanism underlying the response to waterlogging and post-waterlogging reoxygenation in *C. morifolium* remains unknown. In this study, we compared the differences between the transcriptomes of two chrysanthemum cultivars, i.e., the waterlogging-tolerant cultivar “Nannongxuefeng” and the waterlogging-sensitive cultivar “Qinglu”, by performing RNA-seq to elucidate the possible mechanism of waterlogging and reoxygenation in *C. morifolium*. “Nannongxuefeng” had a higher ethylene production under the waterlogging and reoxygenation conditions. Furthermore, the expression of transcription factors and genes that are involved in the hormone response, N-end rule pathway and ROS signaling significantly differed between the two cultivars. “Nannongxuefeng” and “Qinglu” significantly differed in their response to waterlogging and reoxygenation, providing a deeper understanding of the mechanism underlying the response to waterlogging and guidance for the breeding of *C. morifolium*.

## 1. Introduction

Water is crucial for plant growth and development. However, many plants are often subjected to flooding stress due to excess water remaining in soils. Flooding stress is among the most widespread abiotic stressors during the lifespan of plants [1]. Flooding involves the following two major stressors: submergence, which occurs when the entire plant (root and shoot) is under water, and waterlogging, which occurs when only the root-zone of the plant is flooded [2]. The excess water content in the soil replaces the gas spaces surrounding the roots and shoots, thus rendering the plants hypoxic or anoxic [3]. The low oxygen status of plants caused by flooding results in the suppression of various necessary physiological activities, which jeopardizes plant growth and development [4].

When oxygen is limited, aerobic respiration is suppressed in the roots [4]. Anaerobic respiration becomes the primary pathway supplying energy to the plants. Fermentation pathway enzymes, such as pyruvate decarboxylase (*PDC*), alcohol dehydrogenase (*ADH*), and sucrose synthase (*SUS*), are involved in sugar metabolism and are significantly up-regulated during the waterlogging process [5,6,7,8,9]. The limited oxygen also causes the accumulation of reactive oxygen species (ROS), resulting in membrane lipid peroxidation, protein and nucleic acid structure changes, and low antioxidant enzyme activity [10,11,12,13,14]. ROS-scavenging enzymes, such as superoxide dismutase (*SOD*), catalase (*CAT*), and ascorbate peroxidase (*APX*), are impaired under waterlogging stress due to the disturbed balance of ROS generation and removal [15,16,17,18].

Ethylene is a pivotal hormone in the response to waterlogging in plants. The ethylene precursor 1-aminocyclopropane-1-carboxylic acid (ACC) is produced in the plant roots and is transported to the xylem, where it is oxidized to ethylene to mediate waterlogging tolerance in plants [19]. Ethylene production can be detected within several hours of waterlogging. The genes encoding 1-aminocyclopropane-1-carboxylic acid synthase (ACS), 1-aminocyclopropane-1-carboxylic acid oxidase (ACO), and other enzymes that are involved in ethylene synthesis have been shown to be significantly induced following waterlogging stress. For example, in *Arabidopsis thaliana*, waterlogging induces the expression of *AtACS5*, *AtACS7,* and *AtACS8* [8]. Flooding also induces the expression of *RpACS1* in *Rumex palustris*, and enzyme activity assays have shown that the activity of ACS is significantly higher in flooding plants than that in non-flooded plants [20]. However, the induction of ethylene under flooding in *Rumex palustris* can be inhibited by Aminoethoxyvinyl glycine (AVG), which is an inhibitor of ethylene biosynthesis [21].

ERF transcription factors play an important role in ethylene signal transduction [22]. ERF transcription factors belong to a plant-specific transcription factor superfamily related to the stress response [22]. Group VII ERFs are the main transcription factors that are regulating the expression of hypoxia-related genes and the response to waterlogging stress [23,24,25]. The mechanisms of the response to submergence stress in rice, i.e., “escape” and “quiescence”, are regulated by *SNORKEL1* (*SK1*)/*SNORKEL2* (*SK2*) and *SUB1A* and have been extensively investigated [26,27,28,29,30]. In the escape strategy, ethylene signaling induces *SK1*/*SK2* expression and it then activates the gibberellin (GA) pathway to promote the elongation of the rice internode, thus enabling the rice to escape the flooding environment [31]. In the quiescence strategy, *Sub1A-1* is induced by ethylene [32,33] and inhibits the production of ethylene under flooding conditions, thereby inhibiting the elongation growth of the flooded parts [34]. The *Arabidopsis* VII ERFs, including the five members *RAP2.12* (AtERF75), *RAP2.2* (AtERF74), *RAP2.3* (AtERF72), *HRE1* (AtERF73), and *HRE2* (AtERF71) [35], are regulated by the N-end rule pathway, which senses oxygen under hypoxic stress conditions [36]. Transgenic *Arabidopsis* plants with *35S::*RAP2.12 are more tolerant to submergence conditions [37]. Three *Arabidopsis* mutants, i.e., *rap2.12*, *rap 2.2*, and *rap 2.3,* all show a more sensitive phenotype than wild type *Arabidopsis* following submergence stress [37]. Stabilized RAP2 transcription factors can prolong the ABA-mediated activation of a subset of osmotic responsive genes [38]. The constitutive overexpression of HRE1 or HRE2 could rapidly induce hypoxia-responsive genes, such as *PDC1* and *ADH1*, in order to enhance hypoxia tolerance in *Arabidopsis* [39,40].

Reoxygenation refers to the recovery period following the removal of hypoxia stress, and it is closely interrelated with waterlogging stress [2]. However, studies investigating the changes and adaptions in plants in response to post-hypoxia stress are limited. A few studies have explored the mechanisms of the response to post-submergence reoxygenation in plants and revealed rapid changes in transcription, metabolite abundance, and energy production following a shift to normoxic conditions [41,42]. In *Arabidopsis*, hypoxia stress results in a 50% reduction in polysome content, and this change can be reversed by reoxygenation for 1 h [42]. In rice, SUB1A prevents the accumulation of ROS in aerial tissue during drought and desubmergence, thereby improving the survival and rapid dehydration following desubmergence [43]. Reoxygenation conditions result in the rapid accumulation of jasmonates (JAs) and the up-regulation of JA biosynthesis genes in *Arabidopsis* [44]. Moreover, the overexpression of the transcription factor MYC2 enhances tolerance to post-hypoxic stress [44]. Based on the abovementioned results, plants undergo substantial changes and adaptions upon re-exposure to normal oxygen levels, even after a short-term recovery.

Chrysanthemum is a leading ornamental species that is widely popular worldwide [45]. However, most cultivars are susceptible to waterlogging stress and exhibit a wilted, yellow, rotted phenotype under stress conditions. However, a few tolerant cultivars have been identified [46,47]. The morphological, anatomical, physiological, and biochemical responses of chrysanthemum to waterlogging have been described [46,48]. In addition, ethylene production is enhanced by waterlogging in two chrysanthemum cultivars with different tolerance levels, and the tolerant cultivar (“53-4”) has a higher ethylene production than the susceptible cultivar (“13-13”) [46]. Forty-five molecular markers are significantly associated with the waterlogging tolerance of chrysanthemum, and four favorable parental lines have been identified as potential donors for improving waterlogging tolerance [47]. Nevertheless, the molecular mechanism of the response of chrysanthemum to waterlogging is unclear due to its complicated genetic background and the lack of reference genome information. Here, two chrysanthemum cultivars, i.e., “Qinglu” (waterlogging-sensitive) and “Nannongxuefeng” (waterlogging-tolerant), were examined in order to determine the possible difference in their transcriptome profiles in response to waterlogging stress and post-stress reoxygenation and the signaling pathways and regulatory networks that are related to waterlogging and reoxygenation.

## 2. Results

### 2.1. Morphological Changes and Ethylene Measurement in “Nannongxuefeng” and “Qinglu” Following Waterlogging Stress

**Figure 1:**
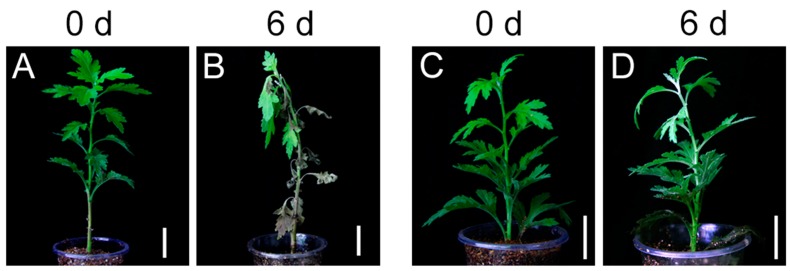
Changes of Phenotypes before and after waterlogging treatment in “Qinglu” and “Nannongxuefeng”. (**A**) Phenotype of “Qinglu” before waterlogging treatment. (**B**) Phenotype of “Qinglu” after 6 d waterlogging treatment. (**C**) Phenotype of “Nannongxuefeng” before waterlogging treatment. (**D**) Phenotype of “Nannongxuefeng” after 6 day waterlogging treatment. Bar = 3 cm.

Here, we compared the morphological changes in two different cultivars, i.e., “Nannongxuefeng” and “Qinglu”, following 6 day of waterlogging stress. No morphological changes were observed in “Nannongxuefeng”, which displayed green leaves and an upright stem (Figure 1C,D). However, “Qinglu” displayed a damaged phenotype with a bent stem and wilted leaves (Figure 1A,B). The mature leaves were completely withered and yellow, and only the young leaves remained partially green (Figure 1A,B). Thus, “Nannongxuefeng” was more tolerant than “Qinglu” to waterlogging stress.

The ethylene production was measured 0 h, 3 h, 6 h, 12 h, and 24 h after waterlogging and after 2 h of reoxygenation after 12 h of waterlogging of the roots. The following four groups were assessed: control, waterlogging, waterlogging + ACC and waterlogging + AVG. The ethylene production in “Nannongxuefeng” was higher than that in “Qinglu” at each time point in all groups regardless of the waterlogging stage or reoxygenation stage (Table 1). During the waterlogging stage, the waterlogging + ACC group had a higher ethylene level than the other groups, followed by the waterlogging group, the control group, and finally, the waterlogging + AVG group at all five time points (Table 1). The same pattern was observed during the reoxygenation stage. Furthermore, the ethylene production peaked at 6 h, declined at 12 h, and dramatically increased at 24 h in both cultivars (Table 1). However, the ethylene production after 12 h + 2 h reoxygenation was higher than that after the 12 h of waterlogging treatment (Table 1). We speculate that the higher ethylene production during waterlogging may be a vital mechanism of the tolerance to waterlogging in “Nannongxuefeng”. The increase in the ethylene production after reoxygenation may be due to the ACC accumulation during submergence in the chrysanthemum roots, and the higher oxygen density promoted the transformation of ACC to ethylene [49].

### 2.2. Transcriptome Sequencing and Read Assembly

To investigate the differences in the mechanisms of the response to waterlogging and reoxygenation between the sensitive and tolerant cultivars, ten libraries from the root tissues of the cultivars, i.e., Q (“Qinglu”) 0 h, QCK (control) 12 h, QW (waterlogging) 12 h, QCK 12 h + 2 h, and QW 12 h + 2 h (waterlogging 12 h followed by 2 h reoxygenation recovery), X (“Nannongxuefeng”) 0 h, XCK 12 h, XW 12 h, XCK 12 h + 2 h, and XW 12 h + 2 h, were generated using Illumina HiSeq. After filtering the raw reads, we obtained 67.01 Mb, 65.06 Mb, 65.43 Mb, 65.43 Mb, 65.97 Mb, 65.48 Mb, 65.48 Mb, 65.02 Mb, 65.52 Mb, and 67.03 Mb of clean reads, containing 6.70 Gb, 6.51 Gb, 6.54 Gb, 6.54 Gb, 6.60 Gb, 6.55 Gb, 6.55 Gb, 6.50 Gb, 6.55 Gb, and 6.70 Gb clean bases, respectively. Of the ten libraries of clean reads, Q20 were above 97.31% and Q30 were above 93.36% (Table 2). The clean read ratios were all above 95.48% (Table 2).

The numbers of transcripts in the libraries from Q 0 h, QCK 12 h, QW 12 h, QCK 12 h + 2 h, QW 12 h + 2 h, X 0 h, XCK 12 h, XW 12 h, XCK 12 h + 2 h, and XW 12 h + 2 h were 154,416, 76,075, 134,241, 149,040, 70,777, 149,070, 151,868, 148,994, 147,947 and 50,049, respectively (Table 3). The total length of the transcripts of the libraries were > 21,171,969. (Table 3). The mean lengths of the libraries were > 423, with N50 > 510, N70 > 320, N90 > 209 and a GC ratio > 39.96% (Table 3).

### 2.3. Gene Annotation and Functional Classification

**Figure 2:**
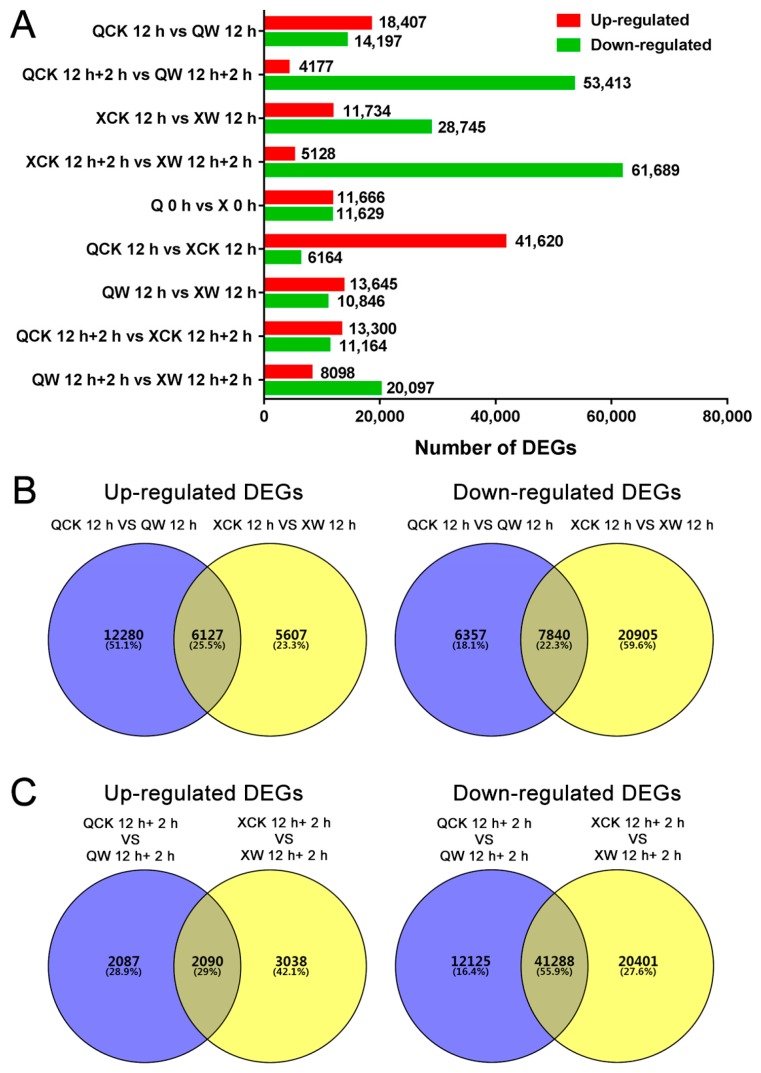
Statistical analysis (**A**) and Venn diagrams (**B**,**C**) of up-regulated and down-regulated differentially expressed genes (DEGs) in response to waterlogging and reoxygenation conditions.

To compare the differentially expressed genes (DEGs) between the two cultivars in response to waterlogging and reoxygenation, we separated the up-regulated and down-regulated genes in the four groups (QCK 12 h vs. QW 12 h, QCK 12 h + 2 h vs. QW 12 h + 2 h, XCK 12 h vs. XW 12 h, and XCK 12 h + 2 h vs. XW 12 h + 2 h) (Figure 2A, Tables S1–S4). The number of up-regulated genes in each group was 18,407, 4177, 11,734, and 5128 (Figure 2A, Tables S1–S4). The number of down-regulated genes in each group was 14,197, 53,413, 28,745, and 61,689 (Figure 2A, Tables S1–S4). In these four comparisons, the tolerant cultivar “Nannongxuefeng” had more DEGs than the sensitive cultivar “Qinglu” during both the waterlogging and reoxygenation stages (Figure 2A, Tables S1–S4), demonstrating that “Nannongxuefeng” had a stronger response to waterlogging and reoxygenation than “Qinglu” at the transcription level. The DEGs in the other five comparisons (i.e., Q 0 h vs. X 0 h, QCK 12 h vs. XCK 12 h, QW 12 h vs. XW 12 h, QCK 12 h + 2 h vs. XCK 12 h + 2 h, and QW 12 h + 2 h vs. XW 12 h + 2 h) are also displayed in Figure 2A. The number of up-regulated genes in each group was 11,666, 41,620, 13,645, 13,300, and 8098 (Figure 2A, Tables S5–S9). The number of down-regulated genes in each group was 11,629, 6164, 10,846, 11,164, and 20,097 (Figure 2A, Tables S5–S9).

Venn diagrams of the abovementioned DEGs during the waterlogging and reoxygenation stages were also performed (Figure 2B,C). In comparison of CK 12 h vs. W 12 h, 6127 up-regulated DEGs were found to be common to both cultivars (Figure 2B). The sensitive cultivar “Qinglu” showed more number of up-regulated DEGs (12,280 genes) when compared to the tolerant cultivar “Nannongxuefeng” (5607 genes) (Figure 2B). Among the down-regulated DEGs, 7840 DEGs were found to be common to both the cultivars (Figure 2B). 6357 DEGs were specifically down-regulated in “Qinglu” and 20,905 were found to be specific to “Nannongxuefeng” (Figure 2B). In comparison of CK 12 h + 2 h vs. W 12 h + 2 h, 2090 up-regulated DEGs were found to be common to both cultivars (Figure 2C). 2087 DEGs were specifically up-regulated in “Qinglu” and 3038 were found to be specific to “Nannongxuefeng” (Figure 2C). Among the down-regulated DEGs, 41,288 DEGs were found to be common to both the cultivars (Figure 2C), suggesting that down-regulated DEGs overlapped greatly under reoxygenation conditions between two caltivars. However, 12,125 DEGs were specifically down-regulated in “Qinglu”, and 20,401 were found to be specific to “Nannongxuefeng” (Figure 2C). The abovementioned results demonstrated that many DEGs varied between the two cultivars in response to waterlogging and reoxygenation (Figure 2, Tables S5–S9). Thus, sensitive and tolerant cultivars may have two independent mechanisms for coping with waterlogging stress and short-term recovery.

To deeply understand the DEGs between the two cultivars in response to waterlogging stress and reoxygenation, the DEGs were further investigated by performing a GO functional analysis. In the Q 0 h vs. X 0 h comparison, 6921 DEGs were assigned a GO term. Nine GO classes significantly fell into the category ‘cellular component’, 8 GO classes significantly fell into the category “molecular function”, and 8 GO classes significantly fell into the category ‘biological process’ (Table S10). In the QCK 12 h vs. XCK 12 h comparison, 16,575 DEGs were assigned a GO term. Twenty-one GO classes significantly fell into the category “cellular component”, 46 GO classes significantly fell into the category “molecular function”, and 33 GO classes significantly fell into the category “biological process” (Table S11). In the QW 12 h vs. XW 12 h comparison, 7041 DEGs were assigned a GO term. Three GO classes significantly fell into the category “molecular function”, and 13 GO classes significantly fell into the category “biological process” (Table S12). In the QCK 12 h + 2 h vs. XCK 12 h + 2 h comparison, 7374 DEGs were assigned a GO term. Fifteen GO classes significantly fell into the category “cellular component”, 52 GO classes significantly fell into the category “molecular function”, and 34 GO classes significantly fell into the category ‘biological process’ (Table S13). In the QW 12 h + 2 h vs. XW 12 h + 2 h comparison, 9443 DEGs were assigned a GO term. Seven GO classes significantly fell into the category “cellular component”, 30 GO classes significantly fell into the category “molecular function”, and 10 GO classes significantly fell into the category “biological process” (Table S14).

A KEGG pathway analysis of the abovementioned DEGs was also performed. In the Q 0 h vs. X 0 h comparison, 7623 DEGs were assigned to 135 pathways in the KEGG database. “DNA replication”, “Isoquinoline alkaloid biosynthesis”, “Tyrosine metabolism”, and “Mismatch repair” were the most significantly enriched terms (Table S15). In the QCK 12 h vs. XCK 12 h comparison, 18,610 DEGs were assigned to 135 pathways in the KEGG database. “Plant-pathogen interaction”, “Plant hormone signal transduction”, “Monobactam biosynthesis”, and “Circadian rhythm–plant” were the most significantly enriched terms (Table S16). In the QW 12 h vs. XW 12 h comparison, 7694 DEGs were assigned to 134 pathways in the KEGG database. “Mismatch repair”, “Homologous recombination”, “DNA replication” and “Nucleotide excision repair” were the most significantly enriched terms (Table S17). In the QCK 12 h + 2 h vs. XCK 12 h + 2 h comparison, 8000 DEGs were assigned to 135 pathways in the KEGG database. The primary terms included “Plant hormone signal transduction”, “Plant-pathogen interaction”, “Flavonoid biosynthesis” and “Isoflavonoid biosynthesis” (Table S18). In the QW 12 h + 2 h vs. XW 12 h + 2 h comparison, 10,028 DEGs were assigned to 135 pathways in the KEGG database. The primary terms included “Plant-pathogen interaction”, “Regulation of autophagy”, “Plant hormone signal transduction”, and “Ubiquitin mediated proteolysis” (Table S19).

### 2.4. Identification of Transcription Factors (TFs) Involved in Waterlogging and Recovery in the Two Cultivars

**Figure 3:**
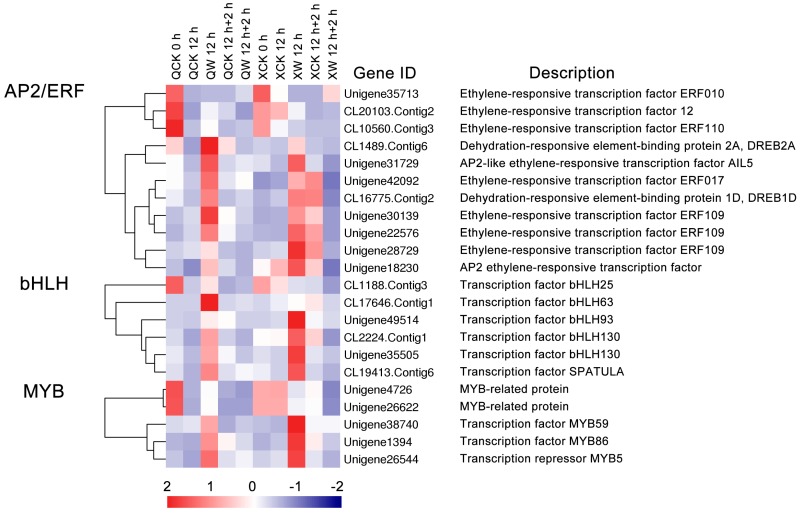
Heatmap analysis of 22 different expressed transcription factors (TFs) from ten libraries. The sample are displayed below each column. Gene ID and annotation of each TF are shown. The expressions of gene are displayed different colors. Red means high expression, and blue means low expression.

Multiple differentially expressed TFs were identified under the waterlogging and reoxygenation conditions. Most TFs belonged to the *AP2/ERF*, *bHLH* and *MYB* families. A heatmap analysis of 11 *AP2/ERFs*, six *bHLHs*, and five *MYBs* was performed (Figure 3). In the *AP2/ERFs* family, *ERF109* (Unigene27829) and *ERF* (Unigene18230) were induced more strongly in “Nannongxuefeng” than in “Qinglu” at 12 h of waterlogging (Figure 3). *DREB2A* (CL1489.Contig6), *AIL5* (Unigene31729), *ERF017* (Unigene42092), *DREB1D* (CL16775.Contig2), and *ERF109* (Unigene30139 and Unigene22576) were more suppressed in “Nannongxuefeng” than in “Qinglu” during the reoxygenation stage (Figure 3). In the *bHLHs* family, *bHLH93* (Unigene49514), *bHLH130* (Unigene35505 and CL2224.Contig1), and *SPATULA* (CL19413.Contig6) were all more up-regulated in “Nannongxuefeng” than in “Qinglu” during the waterlogging stage and down-regulated during the reoxygenation stage (Figure 3). However, *bHLH25* (CL1188.Contig3) and *bHLH63* (CL17646.Contig1) were induced more strongly in “Qinglu” than in “Nannongxuefeng” at 12 h of waterlogging (Figure 3). In the *MYBs* family, *MYB59* (Unigene38740), *MYB86* (Unigene1394), and *MYB5* (Unigene26544) were more up-regulated in “Nannongxuefeng” than in “Qinglu” at 12 h (Figure 3). However, two *MYB*-related genes (Unigene4726 and Unigene26622) were more down-regulated in “Nannongxuefeng” than in “Qinglu” during the reoxygenation stage (Figure 3). The difference in the TF expression mode suggested that those TFs likely played different roles in waterlogging and recovery between the two cultivars.

### 2.5. Hormone Response and Biosynthesis

**Figure 4:**
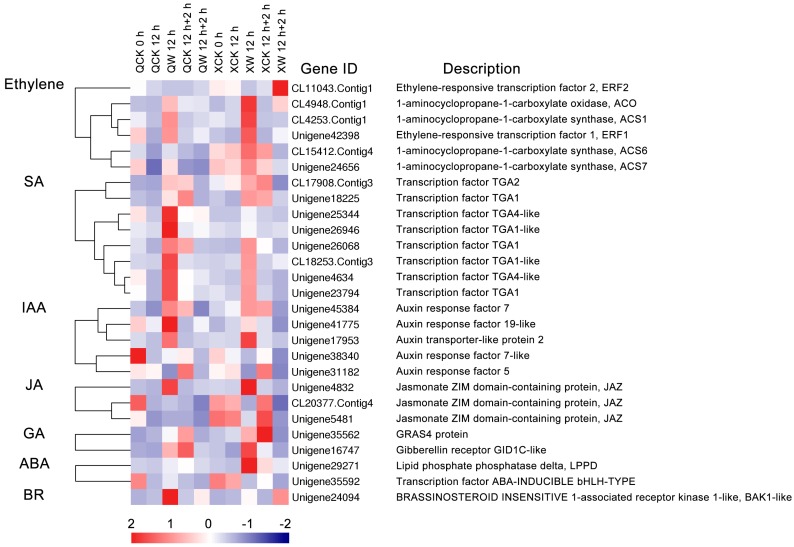
Heatmap analysis of DEGs in roots of “Qinglu” and “Nannongxuefeng” related to hormone response and biosynthesis. The bar represents the scale of the expression levels of each gene (log_2_ FPKM, fragments per kilobase of transcript per million fragments mapped) in each sample, as indicated by red/blue rectangles. Red rectangles represent the high expression of genes, and blue rectangles represent low expression.

Ethylene is a key hormone meditating the response to waterlogging in plants. In this study, two DEGs that were related to ethylene signaling and four ethylene synthesis genes were detected (Figure 4). *ACO* (CL4948.Contig1), *ACS1* (CL4253.Contig1), *ERF1* (Unigene42398), *ACS6* (CL15412.Contig4), and *ACS7* (Unigene24656) were significantly up-regulated at 12 h of waterlogging and down-regulated after 2 h of recovery (Figure 4). In addition, these genes were more induced in “Nannongxuefeng” than in “Qinglu”, indicating that “Nannongxuefeng” had stronger ethylene synthesis activities, which is consistent with the ethylene measurement results (Table 1). These three *ACSs* were down-regulated after 2 h of recovery, but ethylene production increased, which may be due to the reoxygenation. *ERF2* (CL11043.Contig1) was suppressed by waterlogging for 12 h and was up-regulated by reoxygenation for 2 h in “Nannongxuefeng”, while *ERF2* remained stable in the three samples of “Qinglu” (Figure 4), suggesting that *ERF2* may play a different role in the two cultivars in waterlogging and recovery.

In addition to ethylene, DEGs related to other hormones, such as SA (Salicylic acid), IAA (Auxin), JA (Jasmonic acid), and GA (Gibberellin), were also identified. Eight DEGs were involved in SA signaling, and except for *TGA2* (CL17908.Contig3) and *TGA1* (Unigene18225), the other six DEGs were more up-regulated in “Qinglu” than in “Nannongxuefeng” at 12 h of waterlogging stress (Figure 4). Among the IAA-related DEGs, *ARF19*-like (Unigene41775), *ARF7*-like (Unigene38340), and *ARF5* (Unigene31182) were more down-regulated in “Nannongxuefeng” than in “Qinglu” during the reoxygenation stage (Figure 4). Auxin transporter-like protein 2 (Unigene17953) was more up-regulated in “Nannongxuefeng” after waterlogging treatment for 12 h (Figure 4). Two GA-related DEGs, i.e., *GRAS4* (Unigene35562) and *GID1C*-like (Unigene16747), were more up-regulated in “Nannongxuefeng” than in “Qinglu” during the waterlogging stage at 12 h of waterlogging (Figure 4). Three JA-related DEGs (Unigene4832, CL20377.Contig4, and Unigene5481), two ABA- (Abscisic acid-) related DEGs (Unigene29271 and Unigene35592), and one BR- (Brassinosteroid-) related DEG (*BAK1*-like; Unigene24094) were also identified (Figure 4).

### 2.6. Other DEGs Involved in Waterlogging and Recovery in the Two Cultivars

**Figure 5:**
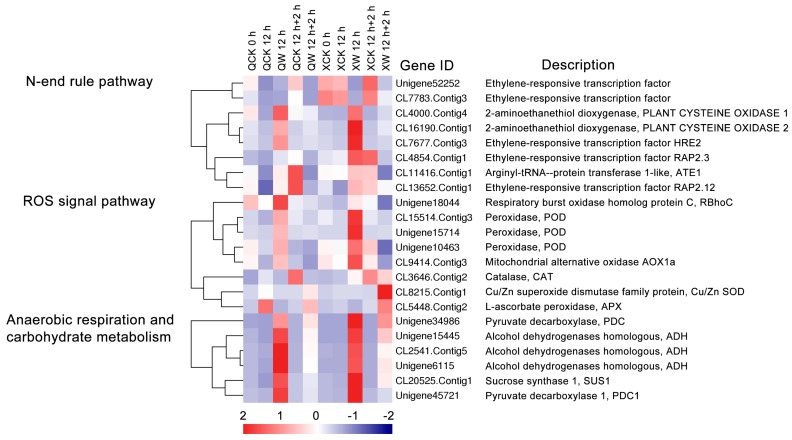
Heatmap analysis of DEGs involved in N-end rule pathway, reactive oxygen species (ROS) signal pathway, anaerobic respiration and carbohydrate metabolism. The bar represents the scale of the expression levels of each gene (log_2_ FPKM) in each sample, as indicated by red/blue rectangles. Red rectangles represent the high expression of genes, and blue rectangles represent low expression.

Group VII ERFs are main transcription factors that regulate the expression of hypoxia-related genes and the response to waterlogging stress via the N-end rule pathway. Here, we analyzed five group VII *ERF*s and other N-end rule pathway genes (Figure 5). Three group VII *ERF*s, *HRE2* (CL7677.Contig3), *RAP2.3* (CL4854.Contig1), *RAP2.12* (CL13652.Contig1), *ATE1*-like (CL11416.Contig1), *PCO1* (CL4000.Contig4), and *PCO2* (CL16190.Contig1) were up-regulated at 12 h of waterlogging stress and down-regulated after 2 h of reoxygenation in “Qinglu” and “Nannongxuefeng” (Figure 5). Except for *PCO1*, the other five gene transcripts were more abundant in “Nannongxuefeng” after waterlogging (Figure 5), thus revealing stronger N-end rule pathway responses in “Nannongxuefeng” than in “Qinglu”. However, another two VII *ERF*s, i.e., *RAP2.12*-like (Unigene52252 and CL7783.Contig3), were continuously down-regulated after waterlogging and recovery (Figure 5). The different expression modes of the group VII *ERF*s indicated that these five members may perform different functions and have different mechanisms in the response to waterlogging.

ROS signaling and changes in homeostasis are among the most vital responses to waterlogging. Here, we found eight DEGs that are related to the ROS signaling pathway (Figure 5). Three *POD*s (CL15514.Contig3, Unigene15714, and Unigene10463) and *AOX1a* (CL9414.Contig3) were more up-regulated by waterlogging of 12 h in “Nannongxuefeng” than in “Qinglu” (Figure 5). Another three DEGs, i.e., *CAT* (CL3646.Contig2), *CSD1* (CL8215.Contig1), and *APX1* (CL5448.Contig2), and were more up-regulated during reoxygenation stage in “Nannongxuefeng” than in “Qinglu” (Figure 5). Thus, the reactive oxygen scavenging enzymes in “Nannongxuefeng” had stronger activities than those in “Qinglu”. In addition, the ROS generating gene *RBohC* (Unigene18044) was more up-regulated in “Qinglu” than in “Nannongxuefeng” during waterlogging stage (Figure 5). The higher up-regulation of the ROS generating genes and lower responses of reactive oxygen scavenging enzymes may exacerbate the sensitivity to waterlogging in “Qinglu”.

A heatmap analysis was also performed to study the DEGs that are related to anaerobic respiration and carbohydrate metabolism in the two different chrysanthemum cultivars during waterlogging (Figure 5). Six key anaerobic respiration genes were found. Two *PDC*s (Unigene34986 and Unigene45721), three *ADH*s (Unigene15445, CL2541.Contig5, and Unigene6115), and *SUS1* (CL20525.Contig1) were significantly up-regulated at 12 h of waterlogging and were down-regulated after 2 h of recovery in the two cultivars (Figure 5). However, the three *ADHs* were more up-regulated in “Qinglu” than in “Nannongxuefeng” during the waterlogging stage (Figure 5). The higher expression of the *ADH*s may accelerate the energy consumption during waterlogging, leading to sensitivity to waterlogging in “Qinglu”.

### 2.7. Differential Transcription of Other Novel Genes Under the Waterlogging and Recovery Conditions

**Figure 6:**
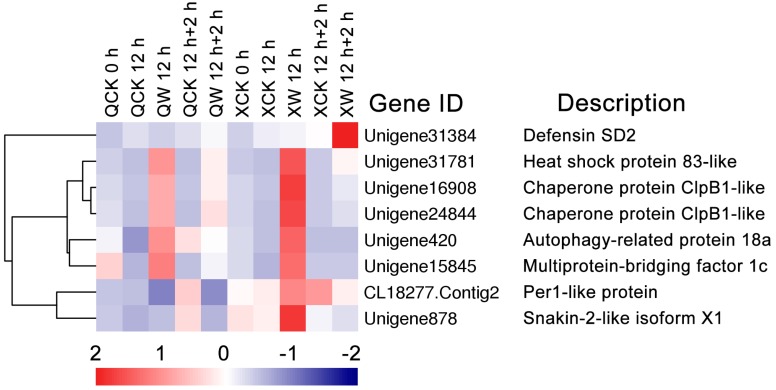
Heatmap analysis of other novel genes under waterlogging and recovery. The bar represents the scale of the expression levels of each gene (log_2_ FPKM) in each sample, as indicated by red/blue rectangles. Red rectangles represent the high expression of genes, and blue rectangles represent low expression.

In addition to abovementioned DEGs, certain novel genes were identified in chrysanthemum under the waterlogging condition. Eight DEGs were further investigated (Figure 6). Per1-like protein (CL18277.Contig2) and Snakin-2-like isoform X1 (Unigene878) were significantly up-regulated in “Nannongxuefeng” at 12 h of waterlogging, but down-regulated in “Qinglu” (Figure 6). Heat shock protein 83-like (Unigene31781), Chaperone protein *ClpB1* (Unigene16908 and Unigene24844), autophagy-related protein 18a (*ATG18a*; Unigene420), and multiprotein-bridging factor 1c (*MBF1c*; Unigene15845) were more up-regulated in “Nannongxuefeng” than in “Qinglu” at 12 h of waterlogging (Figure 6). In addition, Defensin SD2 (Unigene31384) was significantly up-regulated in “Nannongxuefeng” during reoxygenation (Figure 6). However, the functions of these DEGs under the waterlogging and recovery conditions must be further investigated.

### 2.8. Verification of RNA-seq Data by Quantitative Real-Time PCR (qRT-PCR)

**Figure 7:**
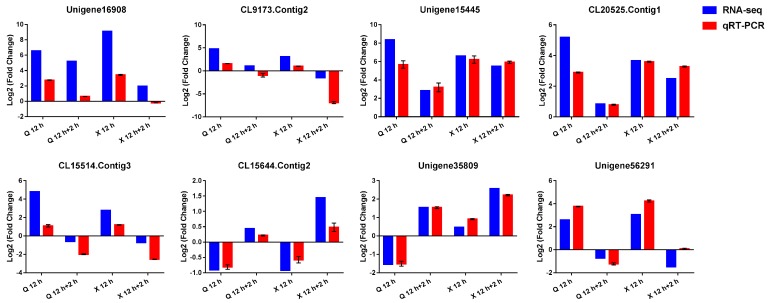
Quantitative real-time PCR (qRT-PCR) validation and RNA-seq data of eight selected DEGs in “Qinglu” and “Nannongxuefeng”. The blue columns represent RNA-seq data and the red columns represent qRT-PCR validation. Three biological duplicates were included for each condition. The y-axis indicates the log_2_-transformed fold change of each DEG under the denoted conditions relative to control. Values of qRT-PCR validation are presented as the log_2_ (Fold Change) ± SE.

To verify the reliability of the RNA-seq data, we selected eight DEGs for further investigation using qRT-PCR methods. These DEGs included chaperone protein *ClpB1* (Unigene16908), two-on-two hemoglobin (CL9173.Contig2), *ADH* (Unigene15445), *SUS1* (CL20525.Contig1), *POD* (CL15514.Contig3), v-type proton ATPase (CL15644.Contig2), ubiquitin-conjugating enzymes (Unigene35809), and a zinc finger protein (Unigene56291). The final qRT-PCR results of the other DEGs were highly consistent with the RNA-seq data (Figure 7), confirming that the RNA-seq data are reliable.

## 3. Discussion

Water is an important environmental factor that is affecting plant growth, morphology, physiological and biochemical metabolism and geographical distribution. However, frequent waterlogging restrains the growth of plants and decreases yield, causing major disasters in agriculture [33]. Therefore, the identification of the plant waterlogging tolerance mechanism has always been an important worldwide agricultural problem. In the present study, we used RNA-seq technology to reveal the transcriptome changes in two chrysanthemum cultivars in response to waterlogging and post-stress reoxygenation. First, we investigated the morphological changes in “Nannongxuefeng” and “Qinglu” after waterlogging stress (Figure 1). After six days of waterlogging stress treatment, “Qinglu” showed a sensitive phenotype with poor growth potential, while “Nannongxuefeng” showed a waterlogging-tolerant phenotype and continued to grow well (Figure 1). Thus, “Nannongxuefeng” is more tolerant than “Qinglu” to waterlogging.

### 3.1. Ethylene Results in Phenotypical Differences Between “Nannongxuefeng” and “Qinglu” after Waterlogging

Ethylene is a vital hormone that is induced by waterlogging stress [19,50]. In this study, the previously reported increase in ethylene production in chrysanthemum after waterlogging was confirmed [46]. We created four treatment groups and detected the ethylene changes during the waterlogging and reoxygenation stages. The waterlogging stress promoted ethylene production in “Nannongxuefeng” and “Qinglu” (Table 1). In addition, the ethylene synthesis precursor ACC combined with the waterlogging treatment promoted greater ethylene production than the waterlogging treatment alone, and the ethylene biosynthesis inhibitor AVG combined with the waterlogging treatment led to a lower ethylene production than the waterlogging treatment alone (Table 1). Interestingly, elevated ethylene production was also observed in both cultivars after two hours of reoxygenation, except for under the control treatment condition (Table 1). The increase in the ethylene production after reoxygenation has been investigated in plants [49]. We speculate that the higher oxygen density results in an increase in ethylene production after reoxygenation. The ethylene production was higher in “Nannongxuefeng” than that in “Qinglu” (Table 1), regardless of the treatment group. The striking ethylene synthesis response may be a vital adaption for coping with waterlogging and reoxygenation in the waterlogging-tolerant cultivar “Nannongxuefeng”.

### 3.2. Changes in the Expression of Transcription Factors under the Waterlogging and Reoxygenation Conditions

Transcription factors are key regulators of the response to various biotic or abiotic stresses [51]. Here, 22 differentially expressed TFs from the *AP2/ERF*, *bHLH*, and *MYB* families were identified (Figure 3). Many studies have shown that these TF families are involved in abiotic stress and they positively improve plant tolerance [24,52,53,54]. The N-end rule pathway plays a crucial role in the sensing of the oxygen density in plants [36,37,55]. The key DEGs in the N-end rule pathway were analyzed in this study. *RAP2.12*, *RAP2.3*, *HRE2, ATE*, *PCO1*, and *PCO2* were up-regulated by waterlogging and down-regulated by reoxygenation in “Qinglu” and “Nannongxuefeng” (Figure 5). The key hypoxia regulators, i.e., *RAP2.12*, *RAP2.3*, and *HRE2,* in “Nannongxuefeng” were more highly induced than those in “Qinglu” (Figure 5), while in *Arabidopsis*, *RAP2.12* and *RAP2.3* are constitutively expressed [23]. In addition, two other group VII *ERF*s, i.e., *RAP2.12*-like (Unigene52252 and CL7783.Contig3), were down-regulated under the waterlogging condition (Figure 5), suggesting that the functions performed by the group VII ERFs, which are the key regulators in the sensing of oxygen in the N-end rule pathway, may explain certain differences between chrysanthemum and *Arabidopsis thaliana* under hypoxic conditions.

Among the other TFs, MaRAP2-4 is a positive regulator of waterlogging tolerance via *Mentha* by regulating the energy-consuming processes and binding the *AtSWEEET10* promoter in *Arabidopsis* [56]. *TaMyb1* (*Triticum aestivum* Myb transcription factor 1) is strongly induced in the roots under the hypoxia condition in wheat [57]. *HYPOXIA RESPONSE ATTENUATOR1* (*HRA1*) is a *trihelix* family TF that is highly up-regulated under hypoxic conditions and it acts as a negative regulator of the key hypoxia transcription factor *RAP2.12* [58]. In addition, *HRA1* negatively regulates the activation of its own promoter [58]. This regulatory mechanism maintains the balance of hypoxia-responsive genes, thereby preventing the excessive accumulation of specific proteins [58]. In this study, *DREB2A*, *ERF017*, *ERF109* (Unigene30139 and Unigene22576), *DREB1D*, *AIL5,* and two *MYB*-related proteins were more strongly suppressed during the reoxygenation stage in “Nannongxuefeng” than “Qinglu” (Figure 3), suggesting that these TFs are negative regulators under reoxygenation conditions. *ERF109* (Unigene27829), *ERF* (Unigene18230), *bHLH93* (Unigene49514) , *bHLH130* (Unigene35505 and CL2224.Contig1), *SPATULA, MYB59*, *MYB86,* and *MYB5* were more strongly induced by waterlogging in “Nannongxuefeng” than in “Qinglu” (Figure 3). These differentially expressed TFs may be regulators that meditate the waterlogging response and stronger tolerance in “Nannongxuefeng”. The functions of these TFs must be further investigated.

### 3.3. Waterlogging and Recovery Lead to Changes in Hormonal Responses and Biosynthesis-Related DEGs

The differential expression of genes that are related to ethylene response and signaling during waterlogging has been reported [59,60]. For example, in a comparative analysis of the transcriptome profiles of two cucumber genotypes (“Zaoer-N”, which is waterlogging-tolerant, and “Pepino”, which is waterlogging-sensitive) under waterlogging conditions, *ACS* and *ACO* were up-regulated in both lines, but the levels were higher in “Zaoer-N” [61]. Here, we selected pivotal genes in the ethylene synthesis pathway, i.e., *ACS1*, *ACS6*, *ACS7,* and *ACO*, and two pivotal genes for ethylene signaling, i.e., *ERF1* and *ERF2*, for further investigation (Figure 4). *ACS1*, *ACS6*, *ACS7,* and *ACO* were more highly induced under waterlogging in “Nannongxuefeng” than in “Qinglu”, suggesting that “Nannongxuefeng” has higher ethylene synthesis activities than “Qinglu” (Figure 4), which is consistent with the ethylene measurement results (Table 1). The *ACS* genes were down-regulated during reoxygenation, while ethylene production was higher after reoxygenation. We speculate that these contradictory phenomena are due to the higher oxygen density caused by the reoxygenation, which promoted the transformation of the remaining ACC to ethylene [49].

Ethylene and IAA are key hormones that are meditating the root growth under waterlogging stress in plants. For example, the accumulation of ethylene promotes the formation of adventitious roots (AR) in waterlogged Rumex plants [21]. Higher accumulation of ethylene in flooded *Rumex palustris* Sm. increases the sensitivity of the root-forming tissues to endogenous IAA, thereby initiating the formation of AR [62]. The treatment with the ethylene biosynthesis inhibitor AVG and the auxin transport inhibitor 1-naphthylphthalamic acid (NPA) can result in a reduction of AR formation in waterlogged *Solanum lycopersicum* [63]. In this study, except for ethylene-related DEGs under waterlogging, several DEGs that are involved in the IAA response were also identified. *ARF7* (Unigene43854), *ARF19*-like (Unigene41775) were more induced in “Qinglu” than in “Nannongxuefeng” during the waterlogging stage (Figure 4). Auxin transporter-like protein 2 (Unigene17953) were more in “Nannongxuefeng” under waterlogging stress (Figure 4). However, *ARF7*-like (Unigene38340) and *ARF5* (Unigene31182) were more suppressed by waterlogging in “Nannongxuefeng” (Figure 4), suggesting that these DEGs may play different roles in Chrysanthemum roots under waterlogging stress. These ethylene and IAA-related DEGs may be the regulators that meditate the root development and AR formation during waterlogging. The functions of these DEGs must be further investigated.

In *Arabidopsis*, *AtERF1* and *AtERF2* are induced by various abiotic stresses, such as drought, salt, and cold [64]. *AtERF1* and *AtERF2* function as transcription activators and bind the GCC-box, thereby meditating down-stream gene expression [64]. In chrysanthemum, *CmERF1* was induced by the waterlogging stress and was suppressed by reoxygenation (Figure 4). However, waterlogging suppressed the expression of *CmERF2* in the two chrysanthemum cultivars. In addition, *CmERF2* was strongly induced during reoxygenation in “Nannongxuefeng” (Figure 4). Thus, *CmERF1* and *CmERF2* likely play different roles than *AtERF1* and *AtERF2* in chrysanthemum under waterlogging and reoxygenation conditions.

The crosstalk among ethylene, JA, SA, and other hormones in response to multiple abiotic stresses has been demonstrated [65,66,67,68]. In this study, DEGs that are related to other hormones were also identified in the two chrysanthemum cultivars, including SA, IAA, JA, GA, ABA, and BR. *TGA2* (CL17908.contig3), *TGA1* (Unigene18225),* GRAS4* (Unigene35562), *GID1C*-like (Unigene16747), and *LPPD* (Unigene29271) were more strongly induced in “Nannongxuefeng” than in “Qinglu” under the waterlogging condition (Figure 4). However, *TGA1* (Unigene23794 and Unigene26068), *TGA1*-like (Unigene26946 and CL18253.Contig3), *TGA4*-like (Unigene25344 and Unigene4634), and *BAK1*-like were more highly induced in “Qinglu” than in “Nannongxuefeng” under the waterlogging condition (Figure 4). A recent study established that *DkTGA1* could be induced by hypoxia and that it exerts a significant influence on the promoters of the de-astringency-related genes *DkADH1*, *DkPDC2,* and *DkPDC3* in Persimmon (*Diospyros kaki* L.) [69]. Thus, these differentially expressed *TGAs* likely play a different role via SA signaling in waterlogging in chrysanthemum. Waterlogging and reoxygenation exert great impacts on the expression of hormone signaling and biosynthesis.

### 3.4. ROS Signaling Pathway, Anaerobic Respiration and Carbohydrate Metabolism

The up-regulation of ROS-scavenging enzyme encoding genes that are caused by waterlogging stress has been reported [70,71]. Here, three *POD*s and *AOX1a* were more significantly induced by waterlogging in “Nannongxuefeng” than in “Qinglu” (Figure 5), suggesting that “Nannongxuefeng” has higher antioxidant enzyme activities than “Qinglu” under waterlogging stress conditions. The level of *RBohC* for ROS generation was much higher under waterlogging in “Qinglu” than that in “Nannongxuefeng” (Figure 5), indicating the presence of higher ROS accumulation in “Qinglu”. The higher ROS-scavenging enzyme activities and the lower ROS accumulation likely contribute to tolerance to waterlogging in “Nannongxuefeng”.

Most environment-induced DNA damages originate in the disruption of ROS homeostasis [72], resulting in DNA single- and double-strand breaks [73,74,75]. For example, a ROS-scavenging enzymes double mutant *apx1/ cat2*, activates the DNA damage response and suffers more than wild type in *Arabidopsis* [76]. In this study, ‘Mismatch repair’, ‘Homologous recombination’, and ‘Nucleotide excision repair’ were significantly enriched in the KEGG analysis of the QW 12 h vs. XW 12 h comparison (Table S17). This result may be involved in the difference of ROS signaling responses between two cultivars. The higher ROS-scavenging enzyme activities during waterlogging may help to reduce the DNA damage in “Nannongxuefeng”.

When hypoxic environments shift to normoxic conditions, significant changes can cause severe damages to plants [77], and ROS accumulation could also occur due to the rapid reoxygenation [13,78,79]. In this study, *CAT, Cu/Zn, SOD,* and* APX* were more significantly induced by reoxygenation in “Nannongxuefeng” than in “Qinglu”, suggesting that certain ROS-scavenging enzymes play a role in the response to reoxygenation. Higher ROS-scavenging enzyme activities during reoxygenation may help chrysanthemum to cope with the damage that is caused by reoxygenation and adapt to environmental changes.

In plants, under hypoxic or waterlogging conditions, anaerobic respiration becomes the major form of ATP generation to cope with the energy crisis that is caused by the low oxygen level [5,7]. Transcriptome analyses of *Cerasus sachalinensis* roots in response to short-term waterlogging have been performed and indicated that the changes in the fermentation mechanism are the first coping mechanisms in response to hypoxic conditions [80]. Representative marker genes related to sucrose synthase and the glycolytic and fermentation pathways, such as *PDC*, *ADH,* and *SUS*, are up-regulated during the waterlogging stage [80]. In this study, *PDC1*, *ADH,* and *SUS1* were significantly up-regulated by waterlogging and down-regulated by reoxygenation in the two chrysanthemum cultivars (Figure 5). We speculate that the changes in the oxygen density may regulate the expression of these genes. Similar patterns in the expression of *PDC1*, *ADH*, and *SUS1* were observed in “Nannongxuefeng” and “Qinglu”, suggesting that the mechanism of anaerobic respiration and carbohydrate metabolism is highly conserved.

### 3.5. Other Genes Involved in Waterlogging and Reoxygenation

Several novel genes with differential expression patterns between the two cultivars under the waterlogging or reoxygenation conditions have also been investigated (Figure 6), including Defensin SD2, *Snakin-2*-like, *HSP83*-like, *ClpB1*, *ATG18a*, and *MBF1c*. Except for Defensin SD2, the other seven DEGs were more highly induced in “Nannongxuefeng” than in “Qinglu”. The heat-inducible transcription factor *HsfA2* is strongly induced by anoxia, and the over-expression of *HsfA2* in *Arabidopsis* seedlings could enhance the tolerance to low oxygen [81]. Here, we found that the heat-related genes *HSP83*-like and *MBF1c* were more strongly induced by waterlogging in “Nannongxuefeng”, but the function of these two genes and the crosstalk between heat and waterlogging stress in chrysanthemum must be further investigated.

**Figure 8:**
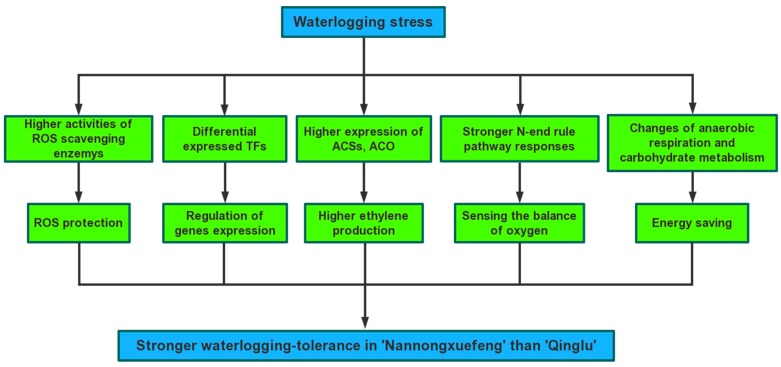
A hypothetical model of responding to waterlogging and reoxygenation in *C. morifolium*.

In conclusion, we used RNA-seq technology to compare the global transcriptomes of two chrysanthemum cultivars in response to waterlogging and post-waterlogging reoxygenation. The DEGs between “Nannongxuefeng” and “Qinglu” varied greatly at the transcription level. In addition, “Nannongxuefeng” produces more ethylene than “Qinglu” under the waterlogging and reoxygenation conditions. Based on these results, a hypothetical model of the response to waterlogging and reoxygenation in chrysanthemum is presented (Figure 8). This study provides a comprehensive transcriptomic analysis for a better understanding of the response mechanism against waterlogging and reoxygenation, and more effective guidance for breeding chrysanthemum.

## 4. Material and Methods

### 4.1. Plant Materials

The “Qinglu” and “Nannongxuefeng” Chrysanthemum cultivars were obtained from the Chrysanthemum Germplasm Resource Preserving Center of Nanjing Agricultural University, China. All of the plants were propagated with cuttings. The medium contained a 2:2:1 (*v*/*v*) mixture of perlite, vermiculite and leaf mold. The rooted seedlings were grown in a greenhouse under constant conditions of 22 °C during the day and a minimum of 15 °C during the night. The relative humidity varied from 70 to 75% (m/m), and no artificial light was provided. The healthy, uniform cuttings were transplanted into a small pot (4 cm down-diameter, 5 cm up-diameter, 9 cm deep) containing a 3:1:1 mixture of garden soil, perlite and vermiculite until the plants developed 10–12 leaves. Then, all of the plants were transferred to the culture room under the 16 h photoperiod (80–100 μmol/m^2^/s illumination) at 22  ±  1 °C conditions for waterlogging treatment.

### 4.2. Waterlogging Treatments

The waterlogging treatment was performed, as previously described by Yin et al. [46]. The pots of chrysanthemums were placed in a 28 cm × 14 cm × 14 cm container filled with tap water, and the water surface was 3 cm above the soil surface. The flooding depth was maintained on a daily basis. Control plants remained well-watered (60% soil moisture) throughout the experiment. The two cultivars were subjected to the waterlogging treatment for 0 d and 6 d for phenotype observation, and 0 h, 12 h, and 12 h + 2 h (2 h reoxygenation) for RNA-seq. Each treatment included at least three replications.

### 4.3. Ethylene Measurements

Four groups of treated chrysanthemum were assessed: control, waterlogging, waterlogging + ACC, and waterlogging + AVG. For waterlogging + ACC and waterlogging + AVG treatments, the pots were subjected in 200 μm ACC solution or 10 μm AVG solution. The treated “Qinglu” and “Nannongxuefeng” root samples were collected at 0 h, 3 h, 6 h, 12 h, and 24 h after waterlogging and after 2 h of reoxygenation after 12 h of waterlogging, and immediately transferred to 22-mL vials and maintained for 6 h at 25 °C. At the indicated time points, 1 mL of headspace air was removed using a syringe and was injected into a GC9790 Plus gas chromatograph (Fuli, Taizhou, China), and the ethylene content was measured, as previously described [82]. At least three independent duplicates were performed. The statistical significance of ethylene measurements data was calculated by One-Way ANOVA method using SPSS 23.0 (SPSS Inc., Chicago, IL, USA). Duncan’s test was applied to assess the differences between treatments.

### 4.4. RNA Extraction, cDNA Library Construction and Sequencing

The abluent “Qinglu” and “Nannongxuefeng” root samples were immediately collected at 0 h, 12 h, and 12 h + 2 h, snap-frozen in liquid nitrogen and stored at −80°C. Each sample was 0.5 g and three biological replicates were performed. The total RNA was isolated from each sample using the RNAiso Plus reagent (TaKaRa Bio, Tokyo, Japan) following the manufacturer’s protocol. The quality and concentration of each RNA sample were verified using an ND-1000 Spectrophotometer (NanoDrop, Wilmington, DE, USA), and only RNA samples delivering the OD260/280 ratio of 1.8–2.1 and the OD260/230 ratio > 1.8 were retained. A 3 μg pool of RNA was formed by combining 1 μg from each biological replicate that was used for the construction of libraries.

The total RNA was treated with DNase I (TaKaRa Bio, Tokyo, Japan), and Oligos (dT) were used to isolate the mRNA. The mRNA was fragmented using a fragmentation buffer. Then, the first cDNA strand was synthesized using the mRNA fragments as templates, which was achieved by random hexamer priming. The second cDNA strand was synthesized by a reaction that was driven by DNA polymerase I (TaKaRa Bio, Tokyo, Japan). The short double-stranded DNA (dsDNA) fragments were purified using a QiaQuick PCR Extraction Kit (Qiagen, Hilden, Germany) and resolved with EB buffer for the end repair and single nucleotide A (adenine) addition. Sequencing adapters were ligated onto dsDNAs and suitable fragments were selected for the PCR amplification.

During the quality control steps, an Agilent 2100 Bioanalyzer and ABI StepOnePlus Real-Time PCR System were used for the quantification and qualification of the sample library. The libraries were paired-end sequenced using an Illumina HiSeq 2000 system. In total, ten sets of raw reads corresponding to Q (“Qinglu”) 0 h, QCK (control) 12 h, QW (waterlogging) 12 h, QCK 12 h + 2 h and QW 12 h + 2 h (waterlogging 12 h followed by 2 h reoxygenation recovery), X (“Nannongxuefeng”) 0 h, XCK 12 h, XW 12 h, XCK 12 h + 2 h, and XW 12 h + 2 h were obtained.

### 4.5. Transcriptome Data Processing and de novo Assembly

Clean reads were obtained from the raw reads after filtering the low-quality reads, adaptor-polluted reads, and reads with a high content of unknown bases (N). After the filtering, the remaining clean reads were stored in the FASTQ format. The de novo assembly using the clean reads was performed using Trinity to obtain the unigenes. Using BLAST+ (v2.2.23) software (http://blast.ncbi.nlm.nih.gov/Blast.cgi) by default parameters, the unigenes were assigned functions based on the homologs present in the NR (www.ncbi.nlm.nih.gov/refseq), NT (www.ncbi.nlm.nih.gov/nuccore), Swiss-Prot (www.uniprot.org), KEGG (www.genome.jp/kegg/kegg1.html), COG (www.ncbi.nlm.nih.gov/COG/), and GO (geneontology.org) databases [83]. Using BLAST2GO (v2.5.0) software (https://www.blast2go.com) by default parameters with NR annotation to get the GO annotation [84]. The expression levels of the unigenes were calculated using the FPKM (fragments per kilobase of transcript per million fragments mapped) method [85]. The DEGs were defined according to an FDR ≤ 0.001 and a |log_2_ fold change| ≥ 1, based on the PossionDis method [86]. The datasets of transcriptome are available in the NCBI repository, Accession No. for library SRP131844.

### 4.6. qRT-PCR Validation and Analysis

Eight DEGs were selected from all DEGs to validate the reliability of the libraries. The primers that were used for these validations were designed using Primer 5.0 software, and qRT-PCR was performed, as previously described by Ren et al. [87]. The chrysanthemum *EF1α* gene (GenBank accession number KF305681) was used as a reference, and the gene primers are listed in Table S20. Three biological replicates were performed per sample.

## Figures and Tables

**Table 1:** Measurement of ethylene production in “Qinglu” and “Nannongxuefeng” at 0 h, 3 h, 6 h, 12 h, 24 h, and 12 h + 2 h recovery. ACC, 1-aminocyclopropane-1-carboxylic; AVG, Aminoethoxyvinyl glycine. Values are presented as the mean ± SE. Letters next to the numbers indicate significant differences between the respective values (*p* < 0.05).

Treatment	Ethylene Production (pl·fw^−1^·h^−1^)					
	Time (Hours)					
	0 h	3 h	6 h	12 h	24 h	12 h + 2 h
”Qinglu”						
Control	1.49 ± 0.22 a	1.28 ± 0.12 c	1.74 ± 0.36 c	1.14 ± 0.05 c	1.48 ± 0.45 c	1.43 ± 0.08 b
Waterlogging	1.49 ± 0.22 a	2.56 ± 0.10 b	2.75 ± 0.11 b	2.3 ± 0.18 b	4.53 ± 0.23 b	1.90 ± 0.57 b
Waterlogging + ACC	1.49 ± 0.22 a	27.67 ± 2.86 a	39.30 ± 2.39 a	23.16 ± 1.57 a	73.78 ± 1.72 a	55.89 ± 3.38 a
Waterlogging + AVG	1.49 ± 0.22 a	0.46 ± 0.02 d	0.37 ± 0.01 d	0.31 ± 0.02 d	1.24 ± 0.15 c	0.88 ± 0.05 c
“Nannongxuefeng”						
Control	2.35 ± 0.32 a	2.19 ± 0.19 b	3.33 ± 0.60 c	2.48 ± 0.31 b	2.40 ± 0.17 d	2.19 ± 0.12 c
Waterlogging	2.35 ± 0.32 a	2.93 ± 0.04 b	4.35 ± 0.26 b	3.03 ± 0.45 b	17.03 ± 2.37 b	5.98 ± 0.28 b
Waterlogging + ACC	2.35 ± 0.32 a	42.93 ± 0.59 a	51.85 ± 4.43 a	32.83 ± 4.94 a	74.22 ± 0.93 a	62.58 ± 4.24 a
Waterlogging + AVG	2.35 ± 0.32 a	0.79 ± 0.07 c	1.24 ± 0.14 d	0.54 ± 0.07 c	3.61 ± 0.61 c	1.17 ± 0.07 d

**Table 2:** Summary of sequencing reads after filtering.

Sample	Total Raw Reads (Mb)	Total Clean Reads (Mb)	Total Clean Bases (Gb)	Clean Reads Q20 (%)	Clean Reads Q30 (%)	Clean Reads Ratio (%)
Q 0 h	67.41	67.01	6.70	97.31	93.36	99.41
QCK 12 h	68.13	65.06	6.51	97.55	94.19	95.48
QW 12 h	65.86	65.43	6.54	98.02	95.02	99.34
QCK 12 h + 2 h	65.86	65.43	6.54	97.68	94.25	99.35
QW 12 h + 2 h	67.39	65.97	6.60	97.67	94.14	97.9
X 0 h	65.86	65.48	6.55	97.88	94.72	99.42
XCK 12 h	65.86	65.48	6.55	97.93	94.84	99.42
XW 12 h	65.86	65.02	6.50	98.09	95.17	98.72
XCK 12 h + 2 h	65.86	65.52	6.55	98.06	95.1	99.48
XW 12 h + 2 h	68.13	67.03	6.70	97.69	94.18	98.38

**Table 3:** Quality metrics of transcripts.

Sample	Total Number	Total Length	Mean Length	N50	N70	N90	GC (%)
Q 0 h	154,416	91,195,520	590	924	490	239	40.91
QCK 12 h	76,075	39,322,976	516	738	389	220	41.74
QW 12 h	134,241	81,313,892	605	950	511	246	39.97
QCK 12 h + 2 h	149,040	90,100,401	604	956	511	244	40.36
QW 12 h + 2 h	70,777	31,838,872	449	567	327	209	42.13
X 0 h	149,070	91,615,155	614	983	522	245	40.02
XCK 12 h	151,868	94,221,259	620	1002	530	247	39.96
XW 12 h	148,994	87,558,806	587	898	489	241	40.18
XCK 12 h + 2 h	147,947	89,776,249	606	955	517	246	40.21
XW 12 h + 2 h	50,049	21,171,969	423	510	320	209	42.44

## Supplementary Materials

Supplementary materials can be found at http://www.mdpi.com/1422-0067/19/5/1455/s1.
